# Afforestation reshapes wildfire activity across global temperate biomes

**DOI:** 10.1073/pnas.2623623123

**Published:** 2026-11-02

**Authors:** Irene Repeto-Deudero, Alejandro Miranda, Juli G. Pausas, Carlos Hormazábal, Blas Mola-Yudego, Javier Lopatin, Susana Gómez-González

**Affiliations:** ^a^ Departamento de Biología - Instituto de Investigación Vitivinícola y Agroalimentaria, Universidad de Cádiz, Puerto Real 11500, Spain; ^b^ School of Forest Sciences, University of Eastern Finland, Joensuu FI-80100, Finland; ^c^ Center for Climate and Resilience Research CR(2), Santiago 8370451, Chile; ^d^Laboratorio de Observación de la Tierra y Ecología de Bosques, Temuco 4811230, Chile; ^e^ Departamento de Ciencias Agronómicas y Recursos Naturales. Universidad de La Frontera, Temuco 4811230, Chile; ^f^Centro de Investigaciones sobre Desertificación, Moncada 46113, Spain; ^g^ Facultad de Ingeniería y Ciencias, Universidad Adolfo Ibáñez, Santiago 7760599, Chile; ^h^ Data Observatory Foundation, Agencia Nacional de Investigación y Desarrollo No. DO210001, Santiago 7510277, Chile

**Keywords:** climate change, wildfire, planted forest, ecosystem vulnerability

## Abstract

In temperate ecosystems, afforestation is rapidly expanding as a flagship climate mitigation strategy. Yet its impact on wildfires remains poorly understood, raising concerns that establishing planted forests over nonforest areas may intensify wildfire activity under increasingly fire-prone climatic conditions. Here, we combine land-use change data, fire activity records, climatic and anthropogenic covariates to quantify how afforestation alters fire occurrence and burned area across 67,248 patches distributed throughout global temperate biomes using satellite records starting in 2001. We found that, on average, afforestation increased fire occurrence by 26% and burned area by 23% in global temperate regions. However, these effects differed markedly among biomes. Wildfire responses ranged from a near-threefold increase in fire occurrence in Temperate Conifer Forests and a 38% increase in Mediterranean Woodlands biomes to an 18% reduction in the Temperate Grasslands, Savannas, and Shrublands biome. In Temperate Broadleaf and Mixed Forests, afforestation reduced fire occurrence but increased burned area, indicating a shift toward less frequent but larger wildfires following the conversion to planted forest. These contrasting, biome-specific responses show that the consequences of afforestation on wildfire activity may depend strongly on ecological and regional context. The strong variability at biome-scale questions the premise that tree planting provides consistent climate mitigation benefits across all landscapes, and highlights the need to evaluate afforestation initiatives alongside regional disturbance regimes. Our findings indicate that wildfire risk should be explicitly accounted for when assessing the long-term effectiveness of afforestation as a climate mitigation strategy in a warming world.

Tree planting is rapidly expanding, both to meet a growing demand for wood products and as a nature-based climate solution (1), with temperate regions identified as high-priority areas (2). However, establishing planted forests on nonforest land (afforestation) may compromise their ecological functions, resilience, and long-term carbon sequestration capacity, and it may instead increase wildfire risk (3). As these initiatives continue to expand, understanding the consequences of afforestation becomes increasingly important, although still poorly resolved.

Anthropogenic climate change is increasing drought, heatwaves, and fuel aridity, creating conditions that favor more frequent and severe wildfires (4–6). These changes have steered extreme fire activity across temperate regions, with major ecological and socioeconomic impacts already observed and projected to increase (7–9). In this context, planting trees in nonforest areas can increase fuel loads and connectivity, promoting fire spread and intensity (3, 10). Consequently, whether afforestation can deliver lasting climate benefits under increasingly fire-prone conditions remains uncertain.

Temperate biomes provide an ideal system for assessing these trade-offs, as these regions have experienced increasingly fire-prone weather (4, 11) and are projected to undergo some of the largest increases in wildfire impacts under near-term warming scenarios (8, 9). Moreover, plantations are approximately twice as likely to burn as native temperate forests (12). Monoculture plantations have been associated with particularly extreme wildfires (see e.g., ref. 13), partly due to increased fuel availability and continuity (10, 11). This convergence creates the potential for compound risks, leading to the hypothesis that afforestation will increase wildfire activity in temperate regions (Fig. 1*A*). Here, we aim to quantify how afforestation alters fire occurrence and burned area across global temperate biomes.

**Fig. 1.**
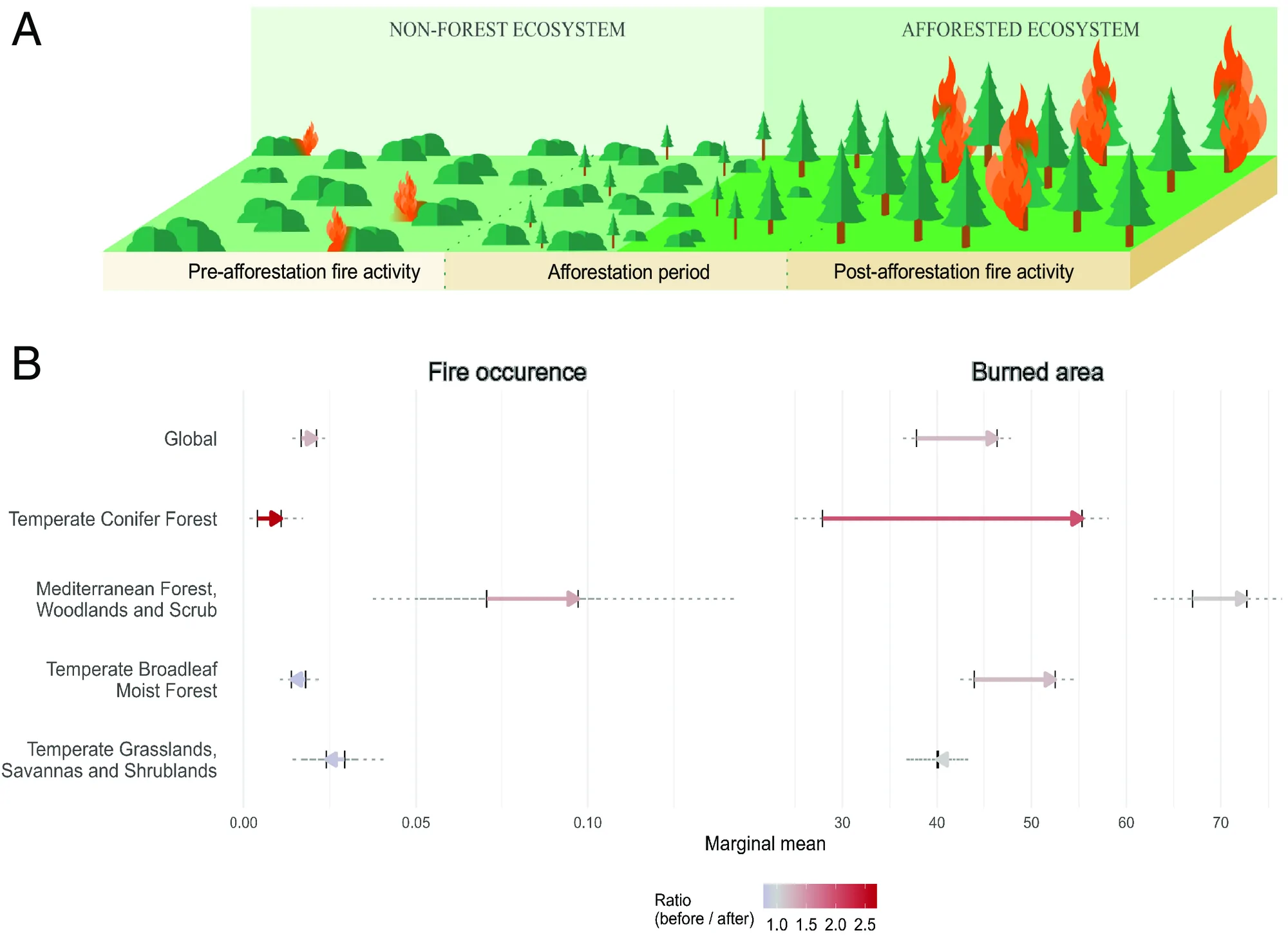
(*A*) Schematic representation of the hypothetical shift in fire activity of nonforest areas following afforestation in temperate regions. (*B*) Effects of afforestation on fire occurrence and burned area across global temperate biomes. For each response, black vertical lines show the estimated marginal means before and after afforestation with 95% CI (horizontal dash lines). Arrows indicate the direction and magnitude of the change. Ratios >1 (forward-pointing arrows) indicate increasing fire activity, whereas ratios <1 (backward-pointing arrows) indicate a reduction.

## Results and Discussion

We identified 67,248 patches across global temperate biomes that transitioned from nonforested land to planted forests between 2001 and 2023, and quantified changes in fire occurrence and burned area before and after this land use change. Both metrics were modeled before and after plantations were established, while accounting for climatic and anthropogenic drivers (*SI Appendix*, *Extended Methods*). We found that fire occurrence and burned area increased with temperature and declined with precipitation, indicating that warm and productive conditions foster fire occurrence as well as larger wildfires. After accounting for these factors, afforestation increased fire occurrence by 26% per hectare per year across global temperate biomes. Standardized to 100 ha over 10 y, absolute probability of fire-occurrence increased from 4.1 to 4.6%. When wildfires arose, expected burned area increased by 23% (Fig. 1*B*).

However, these effects differed markedly among biomes, indicating that regional context strongly influences wildfire activity. The largest changes were found in Temperate Conifer Forests, where afforestation increased fire occurrence by 2.7-fold and approximately doubled the expected burned area (Fig. 1*B*). These landscapes combine high biomass with increasingly extreme fire weather, suggesting that higher fuel connectivity may amplify climate-driven fire activity (11, 12). The Mediterranean Forest, Woodlands, and Scrub biome also showed significant increases in fire occurrence (38%) and burned area (9%) (Fig. 1*B*). In this case, for 100 ha over 10 y, absolute fire occurrence increased from 7.8% to 11.0% after afforestation. In Mediterranean landscapes, seasonal drought, flammable vegetation, and frequent human ignitions favor fire occurrence even before afforestation (10). Therefore, in these already fire-prone, fuel-limited systems, additional biomass from forest plantations may interact with increasingly extreme fire weather to exacerbate wildfire activity (8).

Responses also differed in the remaining temperate biomes, albeit with more moderate absolute changes. In Temperate Broadleaf and Mixed Forests, afforestation reduced fire occurrence (23%) but increased expected burned area by 20% (Fig. 1*B*), revealing a shift toward less frequent but larger wildfires. Since nearly half of the world’s planted forests are located within this biome (14), even modest changes in fire behavior could translate into substantial impacts and socioeconomic consequences. By contrast, Temperate Grasslands, Savannas, and Shrublands were the only biome where afforestation reduced fire occurrence (18%) without significantly increasing burned area (Fig. 1*B*). Given the high natural fire frequency and widespread use of fire across this biome (3, 15), the effects of afforestation are likely to depend on local vegetation, fuel properties, and climatic gradients. Additionally, biome-specific responses may reflect regional differences in ignition sources and fire management, including variation in lightning activity and suppression capacity (10).

**Fig. 2.**
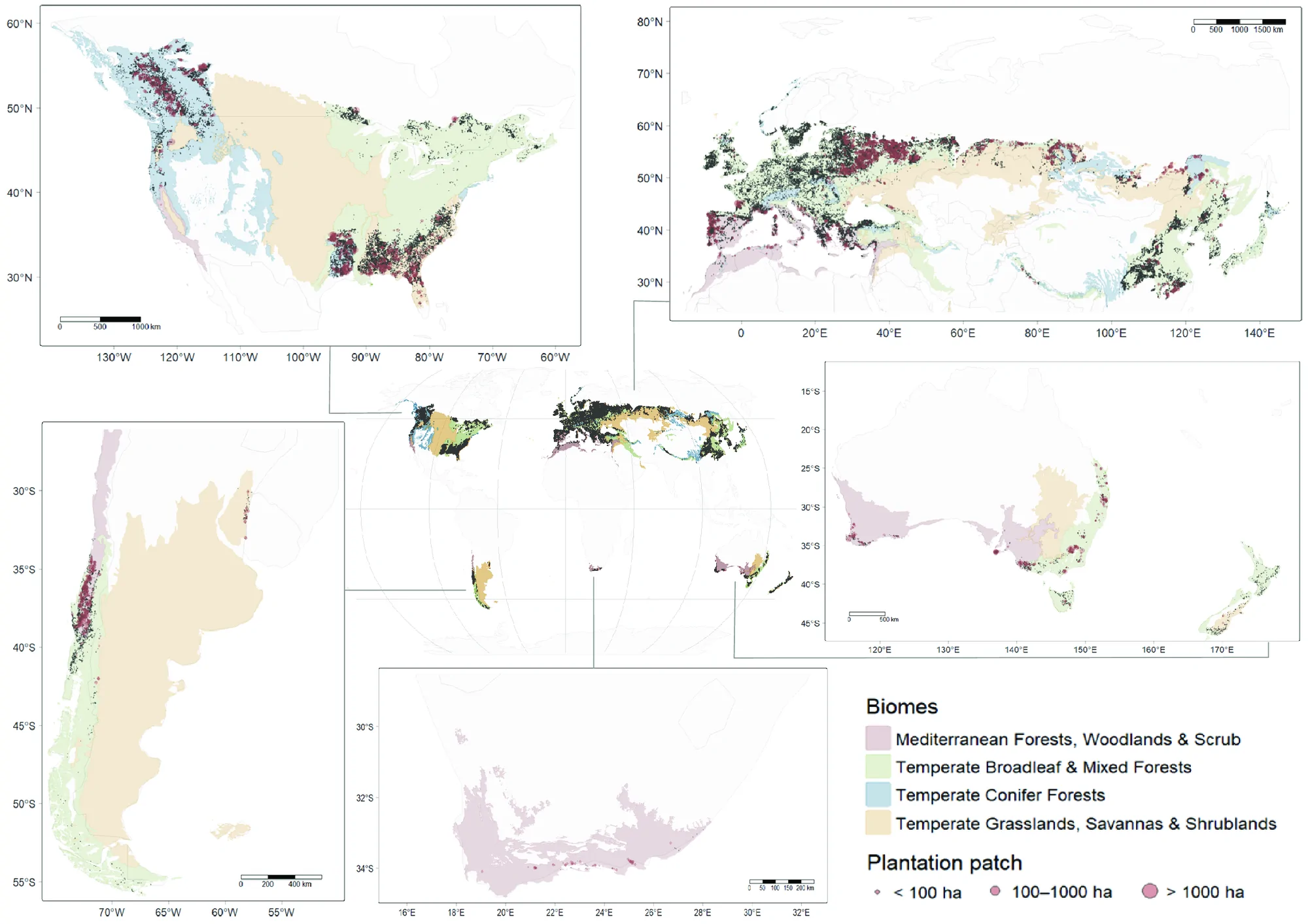
Distribution of nonforest areas converted to planted forest identified across global temperate biomes (central inset, in black). Regional panels display afforested areas (black), highlighting patches where fire occurrence and/or burned area increased after afforestation (in red).

Additionally, we found increases in wildfire activity were clustered in western North America, central Chile, southern Europe, southwestern Australia, and parts of South Africa (Fig. 2), where rapid plantation expansion (14) coincides with increasingly extreme fire weather (4, 7). This convergence suggests that afforestation may further increase wildfire activity in regions where climate already favors extreme fire behavior, with critical socioecological effects (9, 13). Beyond these effects and subsequent risk to human lives (8, 9), exacerbated wildfire activity could have significant consequences for economies relying on the forestry industry, such as Chile and Portugal. Given the contribution of temperate planted forest to global timber production, wildfire-associated timber losses may also have broader implications for future wood supply (2).

Our findings challenge the assumption that tree planting provides consistent climate mitigation benefits, showing instead that afforestation strategies in temperate biomes cannot be planned independently of regional disturbances regime. As afforestation continues to expand as a nature-based solution, potential trade-offs between land-use interventions and changing disturbance regimes should be explicitly considered to ensure their long-term effectiveness under a rapidly changing climate.

## Materials and Methods

We identified afforestation events as transitions from nonforest land to forest using MODIS land-cover data from the last two decades, retaining only areas overlapping with officially mapped planted forests in the SDPT v2 (14). For each afforested patch, we quantified fire occurrence and maximum annual burned area before and after plantation establishment using MODIS burned area records from 2001 to 2023. We used paired mixed-effects models to estimate changes following afforestation while accounting for climatic conditions, accessibility, patch size, and repeated observations of individual patches. Fire occurrence was modeled using a binomial model with a complementary log–log link, whereas burned area was analyzed using a zero-inflated lognormal model. Models were fitted globally and by biome, to quantify variation in afforestation effects across temperate regions (*SI Appendix*, *Extended Methods*).

## Supplementary Material

Appendix 01 (PDF)

## Data Availability

Csv data have been deposited in Zenodo (16).
