# Supplementary material for: Afforestation reshapes wildfire activity across global temperate biomes
Source: Proc Natl Acad Sci U S A. 2026 Nov 2;123(45):e2623623123. doi: 10.1073/pnas.2623623123 (PMC13751663; doi:10.1073/pnas.2623623123)
Supplement: Supplementary file 1 [file pnas.2623623123.sapp.pdf]

PNAS

Supporting Information for

Afforestation reshapes wildfire activity across global temperate biomes

Irene Repeto-Deudero[1,2,3*], Alejandro Miranda[3,4,5], Juli G. Pausas[6], Carlos Hormazábal[4,5], Blas Mola-Yudego[2], Javier Lopatin[3,7,8] & Susana Gómez-González[1,3*]

[1] Departamento de Biología-IVAGRO, Universidad de Cádiz; 11500 Puerto Real, Spain.

[2] School of Forest Sciences, University of Eastern Finland, FI-80100 Joensuu, Finland.

[3] Center for Climate and Resilience Research (CR2); 8370451 Santiago, Chile.

[4] Laboratorio de Observación de la Tierra y Ecología de Bosques, 4811230 Temuco, Chile.

[5] Departamento de Ciencias Forestales. Universidad de La Frontera, 4811230 Temuco, Chile.

[6] Centro de Investigaciones sobre Desertificación (CIDE-CSIC), 46113 Moncada, Spain.

[7] Facultad de Ingeniería y Ciencias, Universidad Adolfo Ibáñez, 7760599 Santiago, Chile.

[8] Data Observatory Foundation, ANID Technology Center No. DO210001, 7510277 Santiago, Chile.

Corresponding authors: * Irene Repeto-Deudero; Susana Gómez-González

Email: irene.repeto@uca.es; susana.gomez@uca.es

This PDF file includes:

Supporting text
SI References

Supporting Information Text: Extended methods

This file contains additional methodological details supporting the analyses presented in the main text. It describes the methodological process behind identification and characterization of afforested areas, fire activity, statistical modelling procedures and limitations.

Characterization of afforested areas

For the purposes of this study, we define afforestation as the establishment of planted forests on previously non-forest land. Here, "non-forest" refers to the categories defined by the MODIS land cover classification starting in 2001 and does not necessarily imply a naturally treeless condition. Accordingly, we focused exclusively on areas undergoing a transition from non-forest land to planted forests. Therefore, reforested areas or naturally regenerating forests were not considered. Newly afforested areas were identified using the *MODIS Land Cover Type Yearly Global 500 m* product (MCD12Q1 v6; **1**), based on *The International Geosphere–Biosphere Programme (IGBP)* (**2**). Land-cover classes were grouped into forest (classes 1–5) and non-forest (classes 6–17), and year-to-year transitions from non-forest to forest were used to detect afforestation events.

To reduce reclassification noise, these transitions were aggregated into three-year windows. We focused on two afforestation periods (2008–2011 and 2011–2014), each framed by comparable pre- and post-transition windows within the *MODIS Land Cover Type Yearly Global 500 m* time series (2001–2023). Selected patches were consistently classified as non-forest prior to transition and forest subsequently. To avoid marginal or unstable classifications, only patches larger than three contiguous pixels (~75 ha) were retained, and areas classified as forest at any available time before the transition were excluded.

To ensure that selected areas corresponded to planted forests, patches were intersected with the most recently available *Spatial Database of Planted Trees* (SDPT v2; **3**), which compiles harmonized spatial information on planted forests reported by 158 countries and captures approximately 90% of global planted forest area (**4**). Tree crops (i.e., rubber, oil palm and orchards) were excluded, retaining only those patches overlapping with mapped forest plantations. The final dataset comprised 30,381 candidate patches afforested during 2008–2011 and 38,426 during 2011–2014 across the global temperate biomes.

Climate variables for pre- and post-afforestation periods were obtained from *ERA5* (**5**). We extracted mean annual temperature, mean annual precipitation and mean maximum temperature of the two warmest months. Human and spatial predictors included minimum Euclidean distance to the nearest road (**6**) as a proxy for human accessibility, and dominant pre-afforestation land cover (MCD12Q1 v6; **1**). Biome and ecoregion classifications followed Dinerstein et al. (**7**), and country boundaries were obtained from (**8**). Each patch was assigned to a unique identifier. All predictors were averaged separately for pre- and post-afforestation periods, resulting in paired observations for each patch (pre vs. post), yielding 134,496 records representing 67,248 afforested patches (global and regional distributions shown in Figure 2, in black).

Fire activity

Fire occurrence and burned area were quantified using the *MODIS Burned Area product* (MCD64A1; **9**) and *MODIS Thermal Anomalies and Fire product* (MOD14A1; **10**). Fire occurrence is here defined as whether an afforested patch experienced at least one recorded fire event during the study period. Accordingly, for each patch and period, fire occurrence was coded as 1 if the burned area was recorded in at least one year, and as 0 otherwise. The burned area was defined as the maximum annual burned area observed within each period. Patches exhibiting fire activity during the transition window itself (for example, 2008–2011) were excluded to avoid confounding land-cover changes with fire activity following plantation establishment.

Statistical analysis

Because fire occurrence was relatively infrequent at the patch level, many patches recorded no fire activity in either period, producing zero inflation, while the relatively small number of large-fire events generated a strongly right-skewed distribution of burned area. We therefore modelled fire occurrence and burned area using the *glmmTMB* package (**11**), which allows flexible specification of generalized linear mixed models and zero-inflated distributions.

Fire occurrence was modelled using a binomial generalized linear mixed model with a complementary log-log link. Burned area was modelled using a zero-inflated lognormal mixed model, separating the probability of zero burned area (zero-inflation component) from the magnitude of burned area when fire occurred (conditional component) (**12**). All numeric predictors were standardized (mean= 0, SD= 1). To account for differences in patch size and exposure time, two offsets were included in all models: log-transformed patch area (as log(area)) and log-transformed duration of the pre- and post-afforestation periods (as log(years)). A random intercept for patch identity accounted for the paired structure of pre- and post-afforestation observations and unmodelled local heterogeneity.

We first fitted global models including climatic, spatial and anthropogenic predictors, biome identity and their two-way interactions. Model selection followed a forward stepwise procedure based on Akaike's Information Criterion (ΔAIC<2; **13**). Once the baseline model structures were established, afforestation status (pre vs. post) and its interaction with biome identity were incorporated to test whether plantation establishment altered fire occurrence and burned area across temperate biomes. Given the strong afforestation × biome interactions, we subsequently fitted biome-specific models using the same predictor structure without terms involving biome identity. This stratified approach allows each biome to have its own baseline fire conditions and afforestation effects to be estimated separately for each biome. Model diagnostics were performed using *DHARMa* (**14**).

To compare fire activity before and after afforestation, we used post-hoc contrasts estimated with *emmeans* (**15**). Both responses of fire activity are expressed as dimensionless ratios, allowing the direction and proportional magnitude of afforestation effects to be compared across responses with different units. For fire occurrence, rate ratios indicate the proportional change in the likelihood of a patch experiencing fire after afforestation. For burned area, ratios indicate the proportional change in expected burned

area when fire occurred. In both cases, ratios >1 indicate an increase, whereas ratios <1 indicate a decrease.

Additionally, to complement the relative post-hoc rate ratios, we estimated absolute probability of fire occurrence before and after afforestation at a common exposure of 100 ha over 10 years, approximating the mean patch size and observation period. Predictions were generated for each patch under both pre- and post-afforestation conditions, while retaining the values for the observed covariates and estimated random effects, then averaged across observations. These describe fire occurrence at a standardized exposure, whereas the post-hoc ratios quantify the proportional effect of afforestation. All analyses were conducted in R (version 4.3.1). Data and code are available at *Zenodo* repository: 10.5281/zenodo.19218387.

Limitations

The MODIS Thermal Anomalies and Fire product (**10**) observations are accompanied by fire radiative power (FRP) data, measuring the energy (megawatts) emitted by a fire at each 1 km pixel where a wildfire is detected. This measure is often used as a proxy of fire intensity (see e.g. **16**). However, only 3,043 (2.3%) afforested patches in our dataset contained valid, non-zero FRP records, since a large proportion of patches recorded no fire events larger than 1 km during the study period. Therefore, we could not include fire intensity metrics into the objectives of our analysis. Further research, potentially targeting large and/or afforested areas exclusively, is needed to evaluate spatial variation in fire intensity within afforested landscapes.

The *SDPT* v2.0 integrates country-reported information that varies in spatial resolution and detail, particularly within the "generic planted forest" category (63% of the database), which may mask differences in species composition and management. The strong contribution of patch-level random effects indicates that local differences, such as lightning ignition efficiency from convective storms or country-specific fire suppression capacity could exert substantial influence on fire activity.

Moreover, the identification of afforestation events is constrained by the temporal coverage of the *MODIS Land Cover Type Yearly Global 500 m product*, which begins in 2001. Consequently, older plantation establishments events are unlikely to be fully captured using this approach. Plantation age could not be consistently determined across our global dataset and was therefore not explicitly included in the models. Fire occurrence in planted forests may, however, vary with time since planting, potentially contributing to explaining the observed variation in fire activity across temperate regions. Our results represent broad global patterns, and subsequent regional analyses will be necessary to disentangle the biome-specific mechanisms that may lie behind these differences, as well as their respective management implications.

SI References

1. M. Friedl, D. Sulla-Menashe, MODIS/Terra + Aqua Land Cover Type Yearly L3 Global 500 m SIN Grid V061. NASA Land Processes Distributed Active Archive Center. https://doi.org/10.5067/MODIS/MCD12Q1.061 (2022).
2. International Geosphere-Biosphere Programme, *The International Geosphere-Biosphere Programme: A Study of Global Change. The Initial Core Project*. IGBP Global Change Rep. 12 (International Geosphere-Biosphere Programme, Stockholm, Sweden, 1990).
3. J. Richter et al., *Spatial Database of Planted Trees (SDPT Version 2.0)*. World Resources Institute. https://doi.org/10.46830/writn.23.00073 (2024).
4. Food and Agriculture Organization of the United Nations, *Global Forest Resources Assessment. Working Paper 188: Terms and Definitions*. https://openknowledge.fao.org/server/api/core/bitstreams/531a9e1b-596d-4b07-b9fd-3103fb4d0e72/content (2020).
5. J. Muñoz-Sabater, ERA5-Land monthly averaged data from 1950 to present. Copernicus Climate Change Service (C3S) Climate Data Store. https://doi.org/10.24381/cds.68d2bb30 (2019).
6. J. R. Meijer, M. A. J. Huijbregts, K. C. G. J. Schotten, A. M. Schipper, Global patterns of current and future road infrastructure. *Environ. Res. Lett.* **13**, 064006 (2018).
7. E. Dinerstein et al., An ecoregion-based approach to protecting half the terrestrial realm. *BioScience* **67**, 534–545 (2017).
8. Esri, *World Countries Generalized*. ArcGIS Hub. https://hub.arcgis.com/datasets/esri::world-countries-generalized/about (2023).
9. L. Giglio, C. Justice, L. Boschetti, D. Roy, MODIS/Terra + Aqua Burned Area Monthly L3 Global 500 m SIN Grid V061. NASA Land Processes Distributed Active Archive Center. https://doi.org/10.5067/MODIS/MCD64A1.061 (2021).
10. L. Giglio, C. Justice, MODIS/Terra Thermal Anomalies/Fire Daily L3 Global 1 km SIN Grid V061. NASA Land Processes Distributed Active Archive Center. https://doi.org/10.5067/MODIS/MOD14A1.061 (2021).
11. M. E. Brooks et al., glmmTMB balances speed and flexibility among packages for zero-inflated generalized linear mixed modeling. *R J.* **9**, 378–400 (2017).
12. A. F. Zuur, E. N. Ieno, N. J. Walker, A. A. Saveliev, G. M. Smith, *Mixed Effects Models and Extensions in Ecology with R* (Springer, 2009).
13. K. P. Burnham, D. R. Anderson, *Model Selection and Multimodel Inference: A Practical Information-Theoretic Approach* (Springer-Verlag, ed. 2, 2002).
14. F. Hartig, *DHARMa: Residual Diagnostics for Hierarchical (Multi-Level/Mixed) Regression Models*, version 0.4.7. https://github.com/florianhartig/DHARMa (2025).
15. R. Lenth, J. Piaskowski, *emmeans: Estimated Marginal Means, Aka Least-Squares Means*, version 2.0.0. https://rvlenth.github.io/emmeans/ (2025).
16. C. X. Cunningham, J. T. Abatzoglou, T. M. Ellis, G. J. Williamson, D. M. J. S. Bowman, Wildfires will intensify in the wildland–urban interface under near-term warming. *Commun. Earth Environ.* **6**, 542 (2025).
